# Enhancing gridded climate products with third party weather data in a rainfall study from Western Australia

**DOI:** 10.1038/s41598-025-11145-0

**Published:** 2025-10-21

**Authors:** Ming Li, Quanxi Shao

**Affiliations:** CSIRO Data61, PO Box 1130, Bentley, WA 6102 Australia

**Keywords:** Rainfall estimation, Quality control, Third-party weather stations, Hydrology, Hydrology

## Abstract

Accurate estimation of weather variables is essential for climate science and real-world applications, yet sparse official weather station networks often limit data reliability in many regions. This study highlights the transformative potential of integrating third-party automatic weather station (TPAWS) data to improve gridded climate data products. Daily rainfall, one of the most important yet challenging weather variables to estimate, is used as a case study in southwestern Western Australia. By incorporating quality-controlled TPAWS observations, we reduce the root mean square error (RMSE) of rainfall estimates by over 15% and false no-rain rates by 30%, with notable improvements during extreme events. These results illustrate how TPAWS data can augment official networks, offering a scalable, cost-effective approach to improve the accuracy of diverse weather variables beyond rainfall alone. Our findings provide compelling evidence of the scientific and practical value of leveraging non-traditional datasets to address data sparsity, opening new avenues for research and development in climate data integration worldwide.

**Supplementary Information:**

The online version contains supplementary material available at 10.1038/s41598-025-11145-0.

## Introduction

Reliable gridded climate products underpin a wide range of applications, from agriculture and water resource management to climate monitoring and weather-related risk assessment, such as insurance. Traditionally, these products are derived from spatial interpolation of point measurements from official weather stations managed by national agencies, such as the Bureau of Meteorology (BoM) in Australia^1,2^. Among these, rainfall stands out as the most difficult variable to estimate due to its high spatial and temporal variability, posing a critical challenge for applications like hydro-climatology^3,4^. Sparse station networks often fail to capture this variability, leading to significant errors^5,6^.

Efforts to improve climate data have increasingly utilized alternative sources, such as satellite and radar-based remote sensing techniques, to supplement official station observations^7–9^. More recently, third-party automatic weather stations (TPAWS) have emerged as a promising resource^10–15^. These stations, often built from inexpensive off-the-shelf devices, provide near-real-time weather data via the internet. In Australia alone, approximately 8,000 TPAWS actively collect rainfall data in real time^16^as opposed to over 700 official BoM automatic rain gauges^17^. These stations fall into two categories: private TPAWS, operated by citizens and shared on online platforms like Netatmo, Weather Underground, and the Weather Observations Website^18,19^, and non-private TPAWS, deployed by entities like local governments or companies for specific purposes. For example, the Department of Primary Industries and regional development (DPIRD) of Western Australia (WA) operates ~ 200 TPAWS (https://weather.agric.wa.gov.au/), while a pilot project from Telstra in Queensland has added 55 TPAWS to enhance weather monitoring capabilities for farming^20^.

Despite their potential, TPAWS data face quality control (QC) challenges due to non-standardized installations, sensor malfunctions, and inconsistent maintenance^21^. Recent studies have developed QC methods tailored to TPAWS observations, focusing on assessing spatial consistency with official observations^22,23^ or within TPAWS networks^24^. Li, et al.^16^ introduced an automated QC procedure that assigns confidence scores to daily rainfall data, enabling users to filter unreliable observations based on risk thresholds. While TPAWS have shown promise—e.g., Bardossy, et al.^24^ reported a 20% reduction RMSE in hourly rainfall estimates in Germany—their broader potential value in improving daily rainfall estimates in regions with sparse observation networks and high rainfall variability (such as Australia) remains underexplored.

**Fig. 1:**
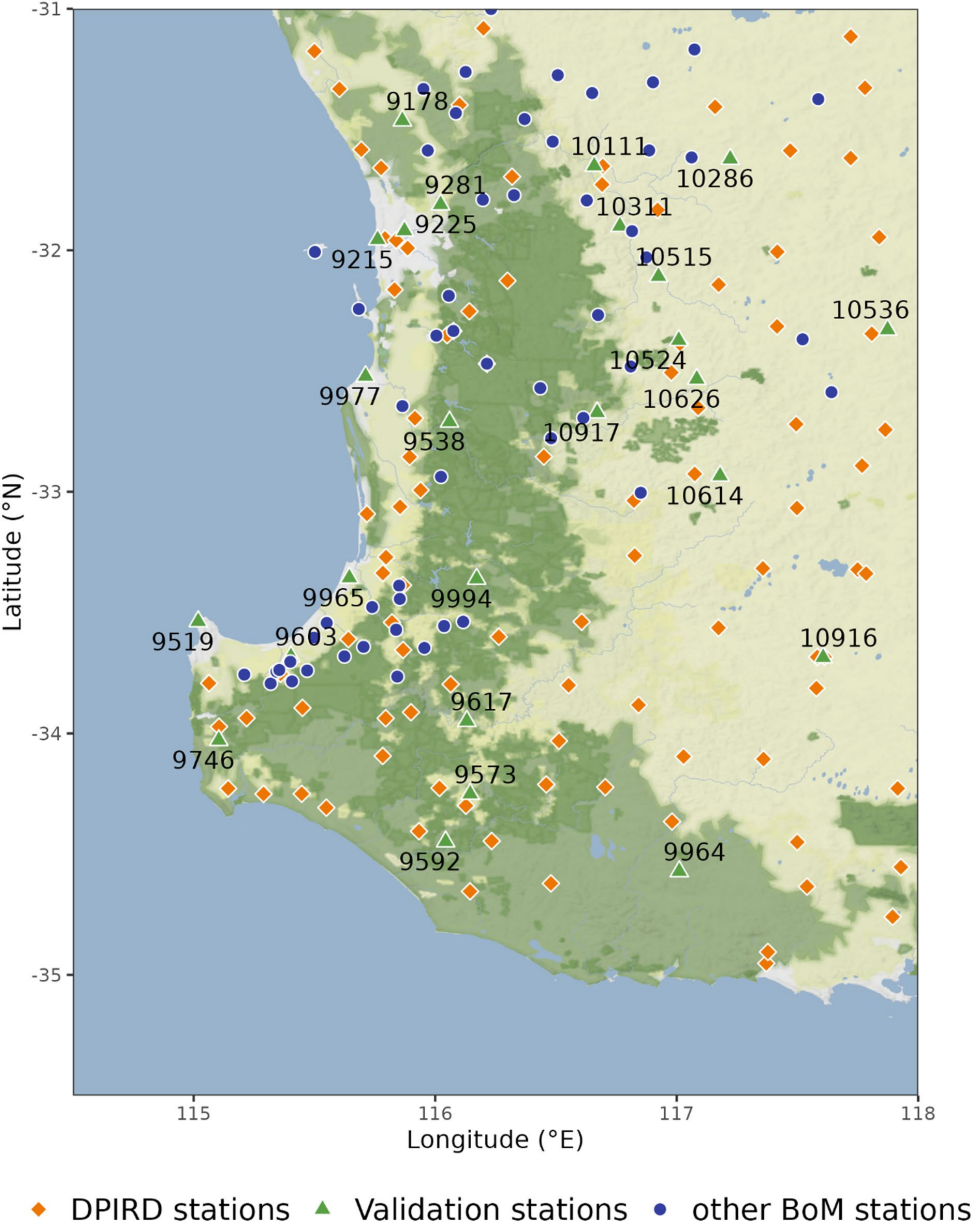
Map of the study area in southwestern Western Australia, showing the locations of validation stations (labelled with station numbers), other BoM stations, and DPIRD stations, each marked with distinct symbols. This map was generated using R (version 4.5.0) with the ggmap package (version 4.0.1). For more details on ggmap, see https://cran.r-project.org/package=ggmap.

A key scientific question is how to effectively utilise these rich TPAWS data, despite QC challenges, to improve the current official climate data products. Unlike prior studies that focus on developing new QC methods or optimizing rainfall estimation techniques, this research uniquely evaluates TPAWS as a novel data source to improve operational products, such as AWAP, in data-sparse regions of Australia. Using daily rainfall estimation in southwestern Western Australia (SWWA) as a case study, this study demonstrates the added value of TPAWS data by applying an established QC method, validated for the study region and data, to improve operational gridded climate products more broadly. This research does not aim to develop new QC methods or identify the optimal rainfall estimation approach but evaluates the contribution of TPAWS data with QC to existing gridded rainfall products. In SWWA, a key agricultural region, 79 BoM stations and 95 DPIRD-managed TPAWS provide rainfall observations. The inclusion of TPAWS has the potential to increases the number of available stations by 120% (Fig. 1). We compared rainfall estimates derived solely from BoM data with those incorporating TPAWS observations, applying the QC method developed by Li, et al.^16^ to ensure data reliability. Our hypothesis is that, with proper QC, erroneous TPAWS data can be treated as outliers, leaving a robust dataset to enhance accuracy. We validated this approach against 25 high-quality BoM stations. These 25 validation stations, also used in Li, et al.^16^, were selected from Australian climate change site networks^25^, with datasets subjected to rigorous quality control and homogenisation techniques. By tackling rainfall—the most difficult variable to estimate—as an example, we highlight TPAWS’ potential to refine estimates of multiple weather variables, offering a scalable framework applicable to other regions and datasets for advancing climate data globally.

## Results

### Overall performance

To evaluate rainfall estimation performance, we estimated daily rainfall at validation stations from 2017 to 2019, comparing four station configurations: (1) BoM only, (2) BoM and DPIRD without QC, (3) BoM and DPIRD with loose QC (threshold 0.0027, three-sigma rule), and (4) BoM and DPIRD with strict QC (threshold 0.01). From totally 99,000 daily observations across 95 DPIRD stations, loose QC removed 850 records, and strict QC excluded an additional 535. Supplementary Figures S1–S2 illustrate the spatial and temporal analysis of DPIRD observations removed by QC. Coastal stations exhibit a higher removal rate, likely due to the challenges of QC under high rainfall variability or sensor malfunctions during extreme weather. Over time, the proportion of removed DPIRD observations has declined, possibly reflecting more frequent maintenance efforts to reduce station malfunctions. Further discussion on QC performance for DPIRD stations is available in Li, et al.^16^. Supplementary Figures S3–S5 depict QC removals for three example rainfall events, elaborated in Sect. 2.3.

Table 1 summarizes error statistics across all validation stations and dates, using BoM-only estimates as the baseline. Incorporating DPIRD data reduced RMSE and MAE by 15–20%, false rainy days by 5–8%, and false no-rain days by ~ 30%. With ~ 70% of observations reporting zero rainfall, improvements were modest overall but substantial for rainy days (≥ 0.2 mm/day), including a 67% reduction in false no-rain errors. Applying QC improved DPIRD data accuracy, with stricter QC generally offering the best performance but also discarding more observations. Balancing accuracy and data retention in QC is challenging. We recommend adjusting QC stringency based on downstream applications. For large-scale spatial and temporal studies, stricter QC should be applied to ensure estimates align smoothly with nearby observations. Conversely, for studies prioritizing localized weather, less strict QC is advised to retain more TPAWS data, preserving fine-scale variability.

**Table 1:** Error statistics for rainfall estimates across all validation stations and dates, with BoM-only estimates as the reference.

	Reference data	RMSE (mm/day)	MAE (mm/day)	False rain rate	False no-rain rate
Reference estimate	BoM	2.132	0.657	0.075	0.085
Test estimate	BoM + DPRID (no QC)	1.800 (− 15.6%, − 16.5%)	0.537 (− 18.3%, − 21.0%)	0.071 (− 5.5%, − 4.4%)	0.058 (− 31.0%, − 67.1%)
	BoM + DPRID (loose QC)	1.767 (− 17.1%, − 18.5%)	0.528 (− 19.7%, − 22.8%)	0.069 (− 7.4%, − 7.8%)	0.058 (− 31.0%, − 67.0%)
	BoM + DPRID (strict QC)	1.762 (− 17.3%, − 18.8%)	0.525 (− 20.0%, − 23.3%)	0.069 (− 7.9%, − 8.6%)	0.059 (− 30.5%, − 66.2%)

Relative improvements, shown in parentheses as percentage differences from the reference, are reported as two values: the first reflects all events, and the second excludes events where reference and test estimates differ by less than 0.2 mm/day.

Table 2 breaks down accuracy for rainy days, grouped into low (0.2–10 mm), medium (10–30 mm), and high (> 30 mm) categories. DPIRD data reduced RMSE by 15–19% across all ranges, with the greatest improvement in low rainfall, followed by medium and high. This reflects the difficulty of estimating extreme rainfall due to its variability. While QC consistently improved accuracy across all categories, strict QC was less effective for high rainfall, likely due to the exclusion of legitimate high-value observations, suggesting a trade-off between quality control and data retention.

**Table 2:** RMSE of rainfall estimates across different observed rainfall ranges, with BoM-only estimates as the reference.

	Reference data	RMSE (mm/day)			
		> 0.2 mm	0.2 ~ 10 mm	10 ~ 30 mm	> 30 mm
Reference estimate	BoM (reference)	4.13	2.51	6.22	14.65
Test estimate	BoM + DPRID (no QC)	3.48 (− 15.9%, − 16.8%)	2.12 (− 15.4%, − 16.8%)	5.15 (− 17.2%, − 18.4%)	12.47 (− 14.9%, − 15.1%)
	BoM + DPRID (loose QC)	3.42 (− 17.2%, − 18.7%)	2.04 (− 18.8%, − 20.7%)	5.15 (− 17.1%, − 18.7%)	12.30 (− 16.0%, − 17.0%)
	BoM + DPRID (strict QC)	3.41 (− 17.4%, − 19.0%))	2.02 (− 19.5%, − 21.5%)	5.14 (− 17.3%, − 19.0%)	12.33 (− 15.8%, − 16.8%)

Relative improvements, shown in parentheses as percentage differences from the reference, are reported for each range.

### Station-wise performance

**Fig. 2:**
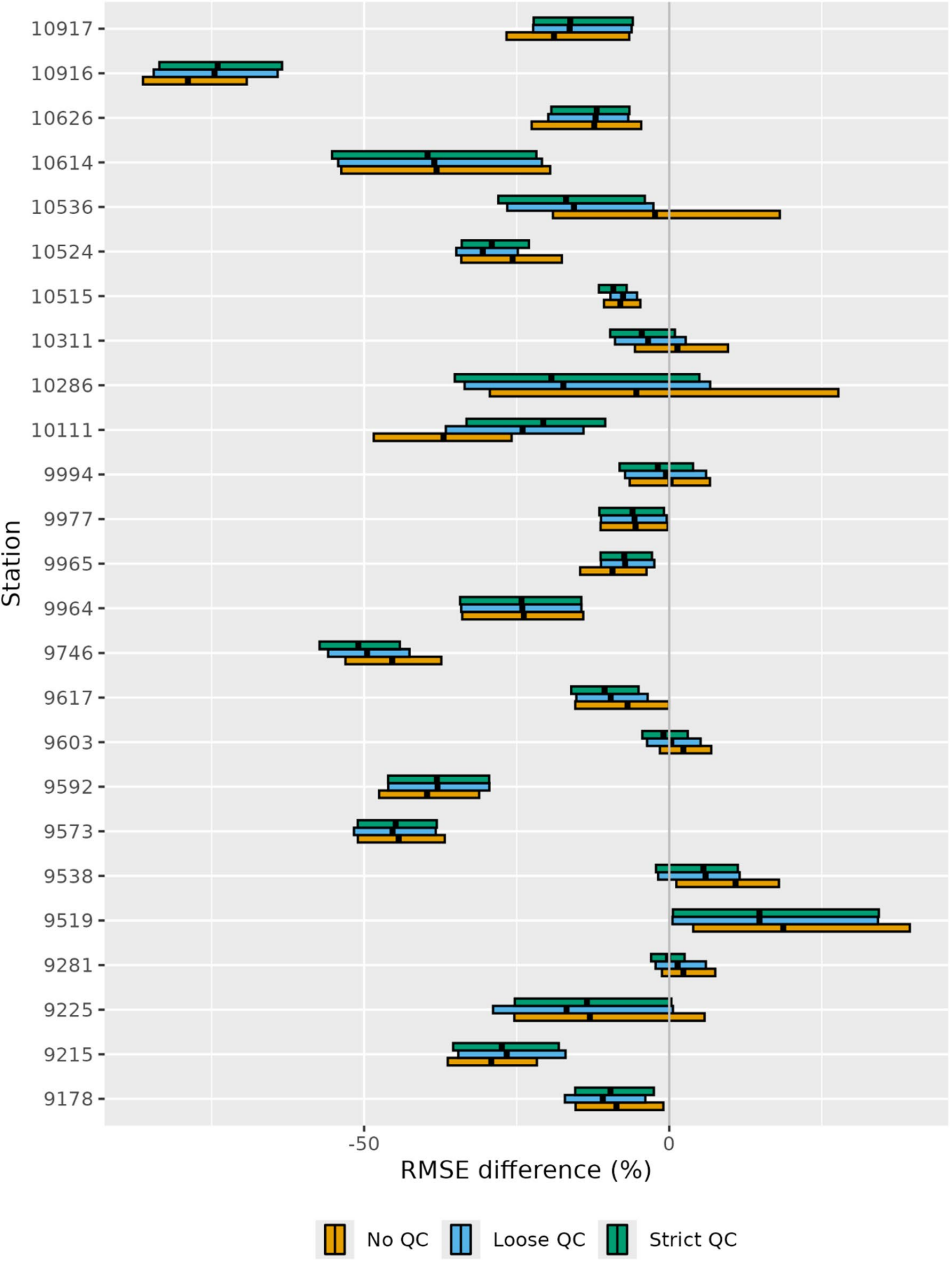
Station-wise RMSE differences between test rainfall estimates and the reference estimate (BoM-only), with 95% bootstrap confidence intervals. Different test configurations are indicated by distinct colours.

Figure 2 illustrates the RMSE differences between test estimates (BoM + DPIRD) and reference estimates (BoM-only) at each validation station. Statistical significance was assessed using 90% bootstrap confidence intervals, derived from 1,000 date resamples with replacement. Adding DPIRD data improved accuracy at most validation stations, with statistically significant gains (upper confidence bound < 0) at 15 of 25 stations (60%). The largest improvement occurred at Katanning (101916), where RMSE dropped by 75%. Isolated from other BoM stations (nearest 86 km away), Katanning benefits from four DPIRD stations within 20 km, including one just 0.6 km away, highlighting TPAWS ability to fill observational gaps.

However, accuracy declined at Cape Naturaliste (9519), with test estimates showing 15–19% higher RMSE—a significant difference. Located on the Leeuwin-Naturaliste Ridge, < 100 m from the Indian Ocean, this station experiences abrupt rainfall from sea breezes. The nearest DPIRD station (26 km south) seems to capture the east-west pattern worse than the nearest BoM station (29 km east), suggesting DPIRD data may introduce noise in coastal microclimates. This opens avenues for refining rainfall estimation by integrating ancillary variables like wind speed and direction.

**Fig. 3:**
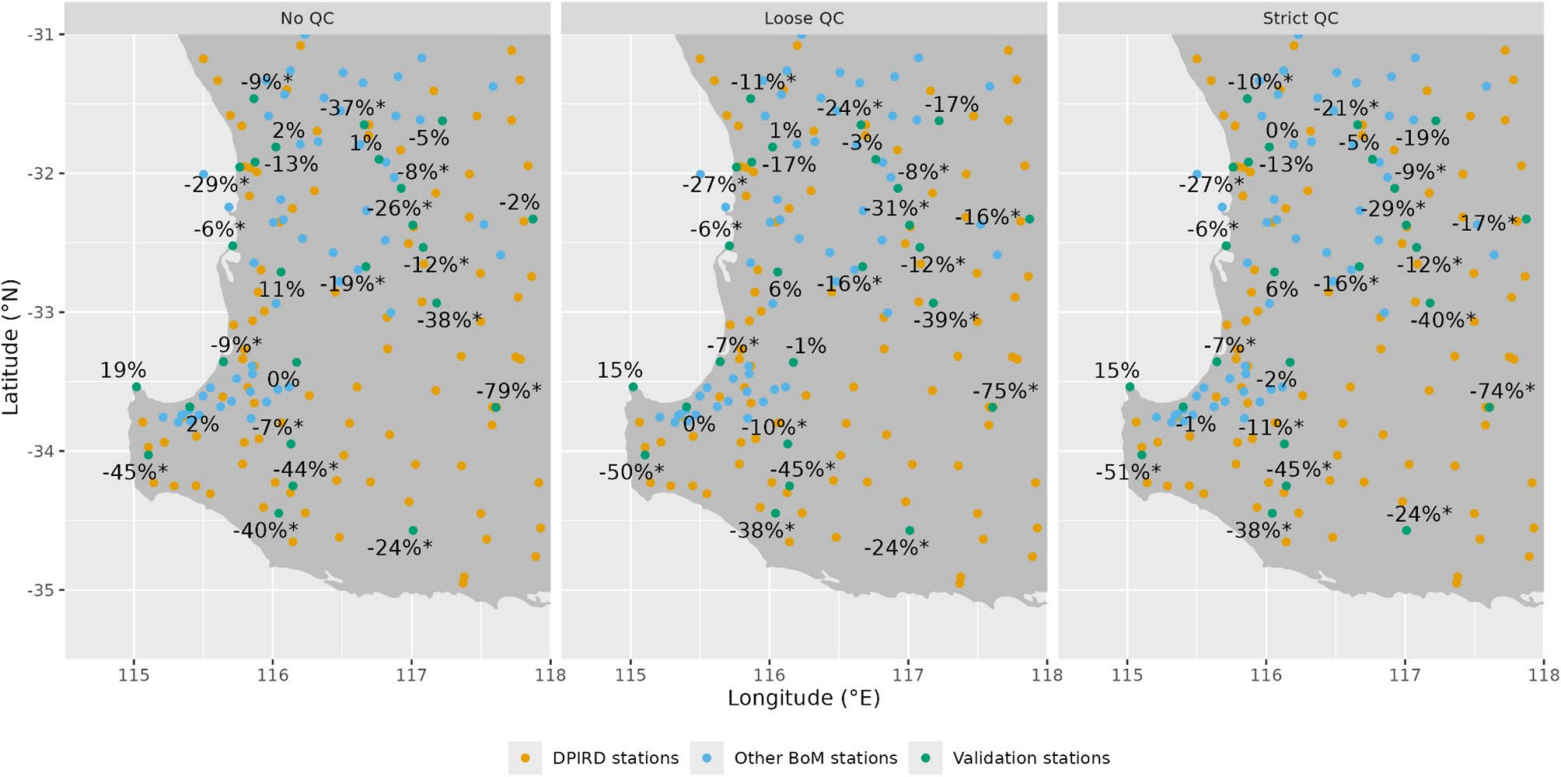
Map of station-wise RMSE differences between test rainfall estimates and the reference estimate (BoM-only). Statistically significant reductions in RMSE are denoted with asterisks.

The effects of QC varied across stations. While QC generally improved accuracy, notable positive effects were seen at stations 10,536 and 10,286, whereas accuracy declined at station 10,111. These variations may stem from localized rainfall events with high spatial and temporal variability. We cautiously interpret these specific cases while still including them in our performance assessment. Figure 3 shows the spatial distribution of RMSE differences across QC configurations, showing greater variability in accuracy improvements inland compared to coastal areas. This pattern aligns with higher rainfall variability near the coastline, which may constrain consistent accuracy gains. Additionally, improvements were more significant in the southern region, where DPIRD station density mitigates the scarcity of BoM stations, compared to the northern region.

### Example rainfall events

We analysed three rainfall events to compare reference and test rainfall estimates, with observations detailed in Supplementary Figures S3–S5.

#### Event 1: 16/01/2018

**Fig. 4:**
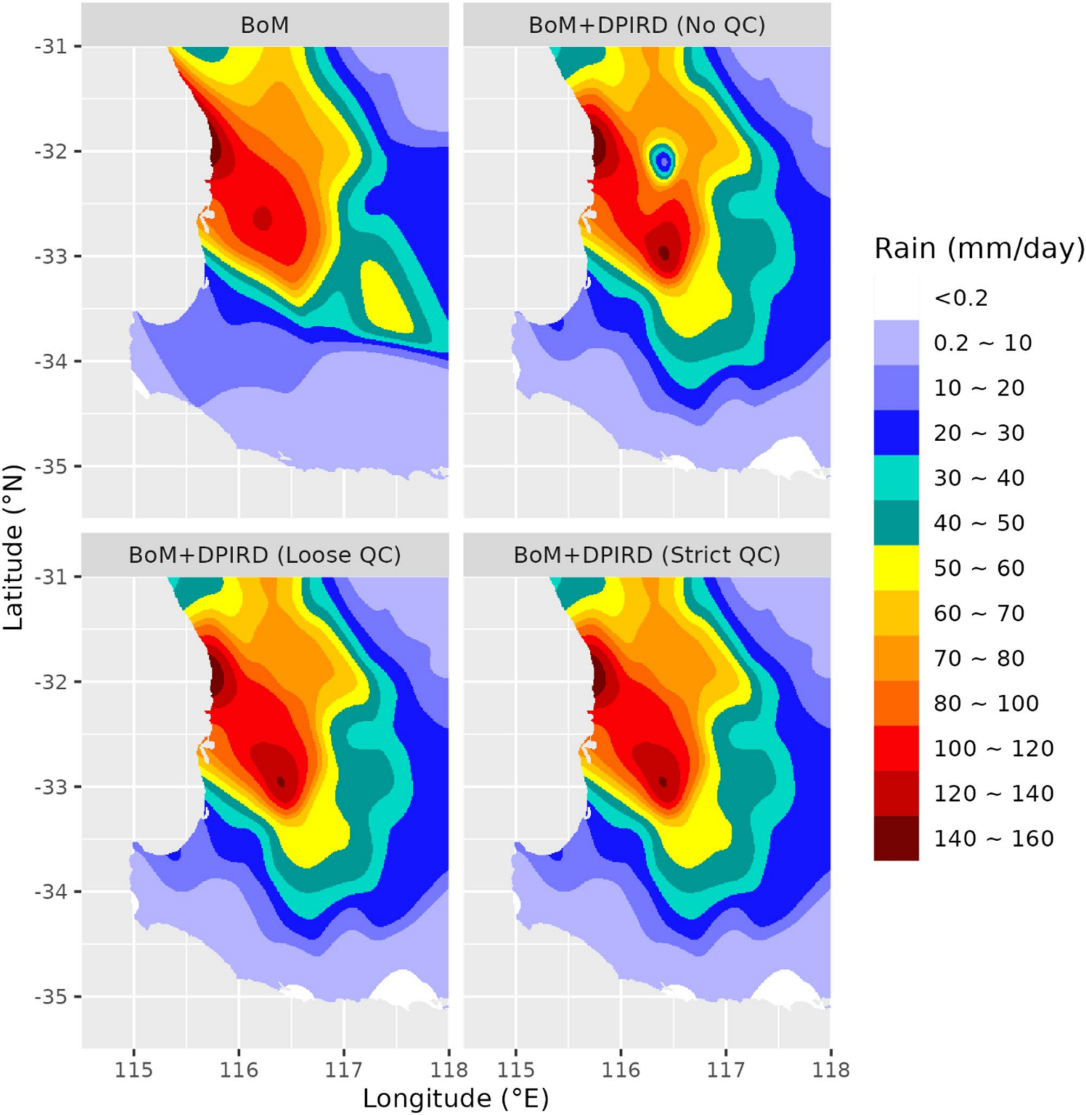
Interpolated rainfall fields at 1-km resolution for 16 January 2018, derived from different rainfall estimates.

This was the heaviest rainfall event in the study period. Observations in Supplementary material Figure S3 show moderate to high rainfall at most stations, with the highest rainfall (> 140 mm/day) recorded in the northwest. Figure 4 compares the interpolated rainfall fields at 1 km resolution, highlighting differences between methods. The reference estimate captured the northwest peak but underperformed in the southeast (< 10 mm, low variability) due to a BoM station malfunction. The test estimates, using DPIRD data, produced a realistic 10–20 mm pattern. A local minima at -32°S, -116.5°E in no-QC estimates (linked to a dubious 12.3 mm DPIRD reading) was removed by loose and strict QC, aligning results with nearby observations.

#### Event 2: 29/01/2017

**Fig. 5:**
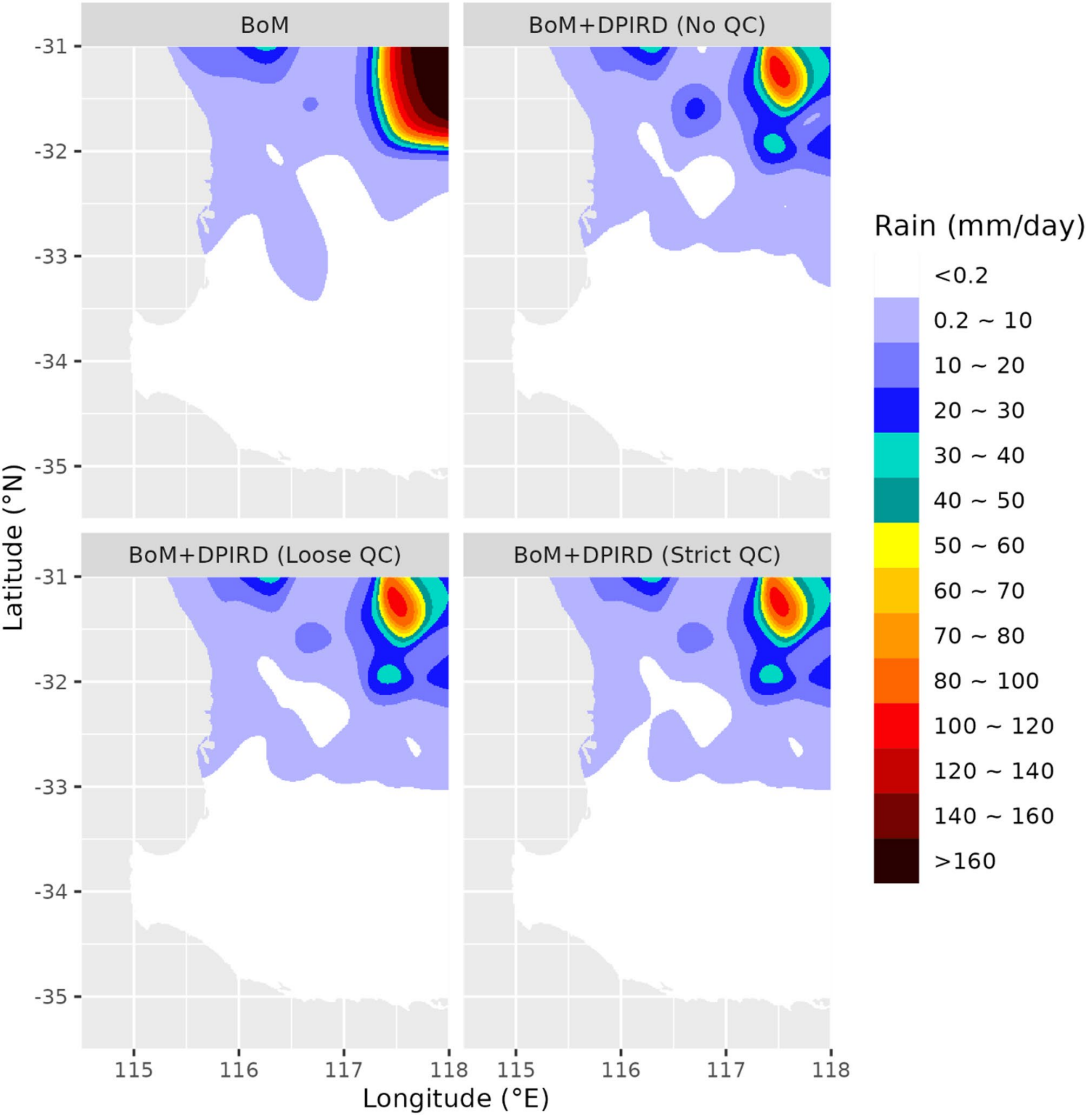
Interpolated rainfall fields at 1-km resolution for 29 January 2017, derived from different rainfall estimates.

This event featured localized heavy rainfall in the northeast, driven by a BoM station (100031) reporting 140.2 mm, an observation inconsistent with surrounding stations. Although quality flags were unavailable, the extreme rainfall reading was likely erroneous, as the official BoM daily rainfall map^26^ has removed this extremely high value through a manual quality control procedure. Figure 5 shows that while the reference estimate highlighted this extreme value, the test estimates, which were tempered by DPIRD data, mitigated its influence and better reflected regional patterns.

#### Event 3: 25/09/2017

**Fig. 6:**
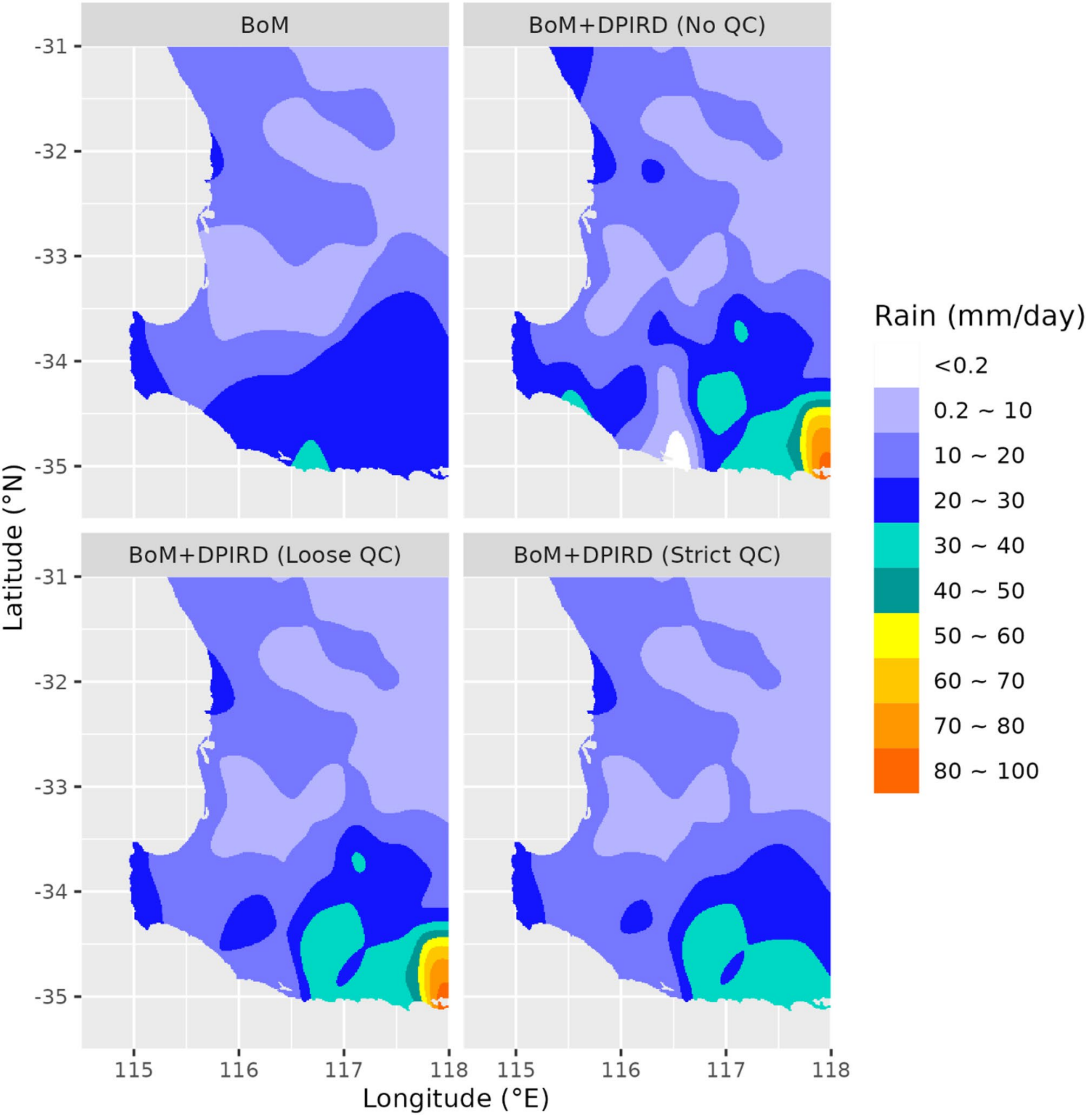
Interpolated rainfall fields at 1-km resolution for 25 September 2017, derived from different rainfall estimates.

This widespread rainfall event affected most BoM and DPIRD stations, with one DPIRD record (67 mm) flagged as suspect by strict QC but not loose QC. Figure 6 shows the reference estimate’s uniform 20–30 mm in the southeast, while test estimates unveiled richer detail (30–40 mm peaks). No-QC and loose-QC estimates emphasized the 67 mm reading, but strict QC smoothed it out. These comparisons highlight the impact of QC stringency on balancing accuracy and variability of rainfall estimates.

## Discussion

**Fig. 7:**
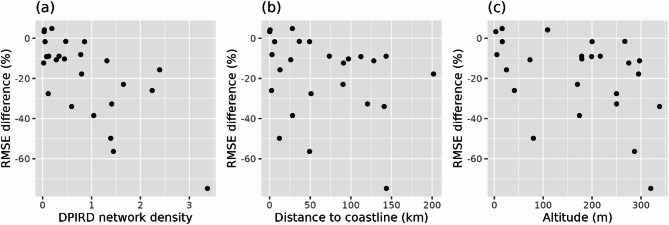
Relationship between RMSE differences (test estimates vs. reference) and (**a**) DPIRD network density, (**b**) distance to the coastline and (**c**) altitude, using strict quality control.

Our results reveal that incorporating DPIRD station observations enhanced rainfall estimation accuracy, with improvements varying across validation stations. Three possible factors drive this variability: DPIRD network density, proximity to the coastline and topography. Figure 7 relates the RMSE difference, between the test estimate with strict QC and the baseline estimate, to these three factors. From Fig. 7(a), network density, calculated as a weighted measure of nearby stations (see Sect. 4.3), indicates that higher DPIRD network density was associated with greater accuracy gains. The most significant accuracy improvements occurred in regions where BoM stations are sparse but DPIRD stations are dense, leveraging the supplementary local weather information provided by additional DPIRD stations to enhance the BoM official network. For example, inland stations like Katanning (Sect. 2.2) benefit from multiple nearby DPIRD stations with no nearby BoM stations, reducing RMSE by up to 75%. Figure 7(b) suggests that distance from the coastline was not a primary driver of average accuracy gains in rainfall estimation, particularly when excluding a notable 74% RMSE reduction at one station, which may be a statistical outlier. However, the variability in accuracy gains was significantly higher for stations within 50 km of the coast compared to those further inland. This pattern aligns with high spatial rainfall variability, where coastal microclimates, as observed at Cape Naturaliste (9519), likely contribute to challenges in achieving consistent accuracy improvements. Thus, high spatial variation, particularly in coastal regions, appears to limit the effectiveness of rainfall estimation approaches. Inland areas, where DPIRD stations are concentrated to serve agriculture, show more consistent improvements. This distribution reflects the network purpose, providing enhanced rainfall estimates particularly valuable for farmers in Western Australian agricultural zones. Although there was some relationship between elevation and the degree of error reduction achieved, the relationship was not strong as seen in Fig. 7(c). It suggests that there was a weak-moderate trend of greater RMSE reductions for validation sites with higher elevations. The strength of the trend may have been affected by the limited number of stations across this large study region. In theory, even slight elevations in coastal areas can influence rainfall patterns by lifting windward moisture, leading to increased rainfall on the windward side and drier conditions on the leeward side. Consequently, topography contributes to increased spatial rainfall variability, meaning the incorporation of additional weather stations in rainfall estimation is more valuable. Topography should be a key consideration when siting new TPAWS stations to enhance rainfall estimation accuracy.

**Fig. 8:**
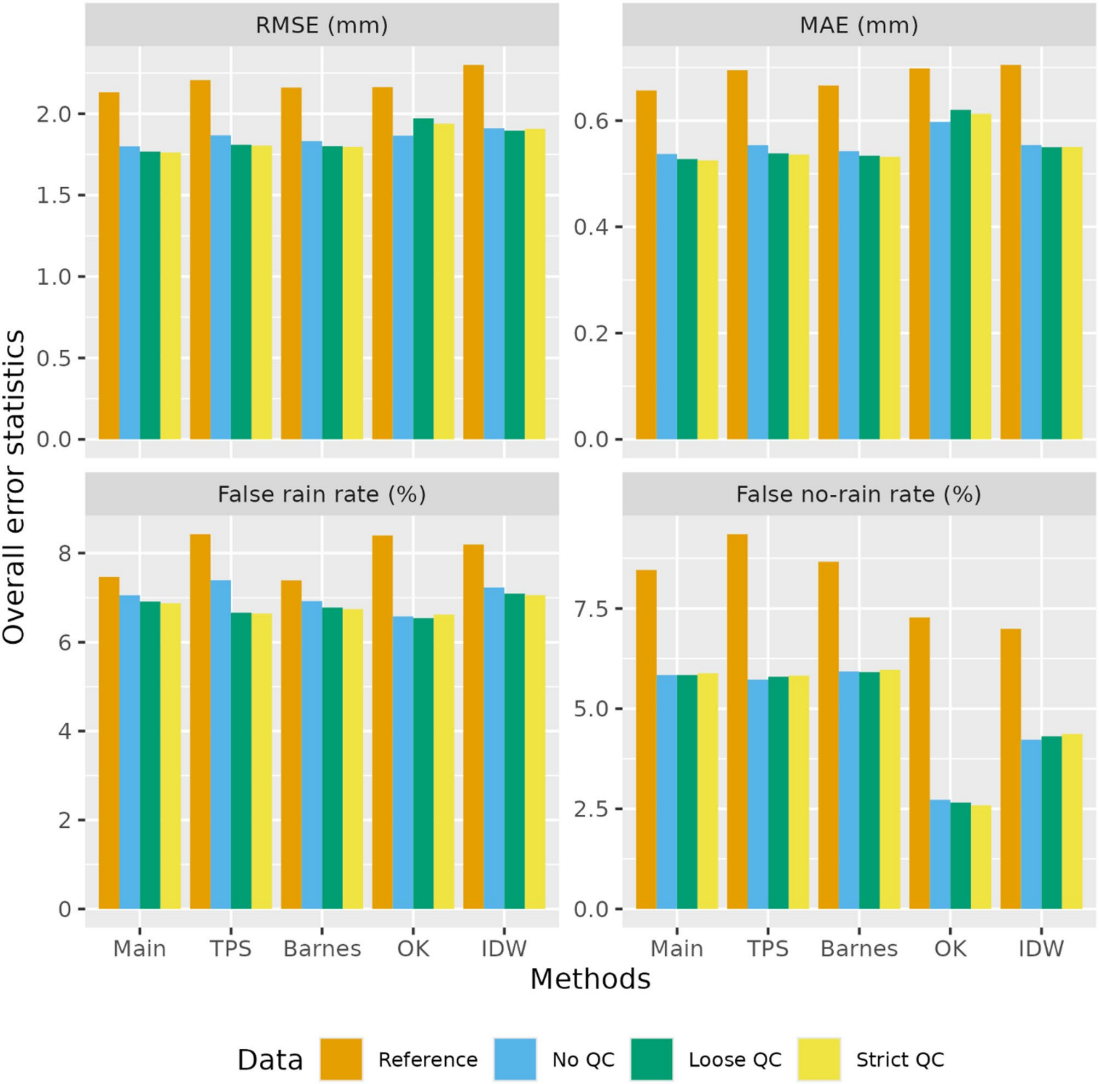
Comparison of overall error statistics for rainfall estimates derived from different geostatistical methods and data configurations.

To evaluate the robustness of the added value of TPAWS observations in improving rainfall estimation, we further examined four traditional geostatistical methods: thin-plate splines (TPS), second-pass Barnes interpolation, ordinary kriging (OK), and inverse distance weighting (IDW). The main method (see details in Sect. 4) applies Barnes interpolation to monthly anomaly data (rainfall divided by its long-term monthly average), whereas the second comparison method applies it directly to the original rainfall data. Figure 8 compares overall error statistics across the same four QC configurations. All methods were improved with DPIRD data, with QC further increasing accuracy by eliminating unreliable records. Improvement magnitude varied across methods, though the trend persisted. This consistency suggests TPAWS data can enhance rainfall estimation broadly, regardless of interpolation technique, though further research should quantify these gains. Similar to the main method, these comparison methods may exhibit varying performance across stations, particularly in areas with low versus high station density. Since this study aims to confirm the added value of TPAWS observations with QC in enhancing the official gridded rainfall estimates in Australia, we do not further analyse the detailed performance of other comparison methods. Future research could conduct regional validation to identify the most critical factors for specific rainfall estimations. Recent studies have developed more advanced algorithms to generate gridded rainfall estimates, such as machine learning-assisted interpolation^27^, data assimilation^28^ and deep learning-based super-resolution methods^29^. Future investigations could explore optimal integration of TPAWS observations to enhance these advanced methods.

Our findings highlight TPAWS potential to advance gridded climate products beyond rainfall. By addressing rainfall, a notably challenging variable to estimate, this study presents a scalable approach for integrating third-party data, applicable to various weather variables in regions with limited official networks. Immediate benefits for agriculture emerge, yet strongly localized spatial variations, such as those observed along the coast in this study, suggest that future enhancements will be valuable, such as incorporating ancillary variables (e.g. wind direction) or optimizing TPAWS placement. With TPAWS networks expanding globally, their integration into climate data frameworks offers prospects for more accurate, locally relevant climate products.

## Methods

### Data quality evaluation procedure

Li, et al.^16^ developed a quality evaluation procedure for daily rainfall observations from TPAWS in Australia, comparing them against two reference datasets: the Australian Gridded Climate Data (AGCD) and Radar Rainfields. The process begins with a domain test to confirm values fall within a plausible range, followed by separate comparisons with each reference source. A confidence score $\:CS$ measures reliability based on the difference between the observed rainfall $\:{R}_{o}$ and the reference rainfall $\:{R}_{s}$:

$$\:CS=\left(1-2\left|{\Phi\:}\left(\frac{f\left({R}_{o}\right)-\mu\:-f\left({R}_{s}\right)\:\:}{\sigma\:}\right)-0.5\right|\right)\times\:100\%$$.

Here, $\:{\Phi\:}$ represents the cumulative distribution function of a standard normal distribution, $\:f$ denotes the log-sinh transformation, and $\:\mu\:$ and $\:\sigma\:$ are the bias and standard deviation parameters, respectively. All model parameters are optimized by the maximum likelihood estimation utilizing a dataset spanning at least two years of historical observations (see Li, et al.^16^ for technical details). A higher $\:CS$ signifies a more reliable observation from TPAWS, with a $\:CS$ of 100% indicating a perfectly reliable observation. If the minimum requirements, as detailed in Li, et al.^16^, are met, we calculate the confidence score for two distinct reference datasets, labelled as $\:C{S}_{AGCD}$ and $\:C{S}_{Rainfields}$. The overall confidence score $\:{CS}_{o}$ is then determined by selecting the maximum value from these individual confidence scores:

$$\:{CS}_{o}=\text{max}\left(C{S}_{AGCD},\:C{S}_{Rainfields}\right).$$

Using a threshold, denoted as $\:{c}_{o}$ for $\:{CS}_{o}$, which reflects the risk preference for specific applications, we classify $\:{R}_{o}$ as an erroneous observation only if $\:{CS}_{o}<{c}_{0}$.

### Daily rainfall estimation

In this study, we estimated daily rainfall by an anomaly-based approach, which is inspired by the geostatistical techniques used to generate daily rainfall analyses in the AGCD dataset^2^. Our approach decomposes of rainfall into a multiplication of its long-term monthly average and an associated ‘anomaly’. The long-term monthly average is obtained directly from AGCD at the closest grid point, and the rainfall anomaly is spatially estimated from the observations from reference stations. The reference stations in this study are based on BoM stations and TPAWS after appropriate QC described in Sect. 4.1. Note that the reference stations for the AGCD dataset include some BoM stations that are not publicly available. Ideally, we would estimate the long-term monthly average rainfall from reference stations using a statistical method, such as the three-dimensional smoothing splines used for AGCD. However, since most TPAWS have less than 10 years of observations, deriving a reliable long-term monthly average based on TPAWS data is challenging, as it typically requires at least 30 years of data.

For a given day, we denote the daily rainfall at location $\:s$ by $\:P\left(s\right)$, which can be decomposed by the following equation:

$$\:P\left(s\right)=M\left(s\right)A\left(s\right)$$,

where $\:M\left(s\right)$ and $\:A\left(s\right)$ are the monthly average rainfall and the corresponding daily anomaly on this target day. We obtain $\:M\left(s\right)$ directly from the long-term monthly average rainfall from AGCD at the closest grid point. Additionally, we impose a minimum threshold of 5 mm for $\:M\left(s\right)$ to ensure the stability of interpolating the daily anomaly, especially during periods of extremely low monthly average rainfall. The daily anomaly $\:A\left(s\right)$ is estimated from a two-pass Barnes interpolation^30^which is also used for estimating daily rainfall anomaly in AGCD. We identify all reference stations within a radius of 200 km from the target location $\:s$, denoted as $\:{s}_{1},{s}_{2},\dots\:,{s}_{n}$. We define the $\:k$-th pass Barnes spatial interpolation, $\:{g}_{k}\left(s\right)$, as

$$\:{g}_{k}\left(s\right)={g}_{k-1}\left(s\right)+\frac{{\sum\:}_{j=1}^{n}v\left({d}_{j}\right)\left\{{g}_{k-1}\left({s}_{j}\right)-A\left({s}_{j}\right)\right\}}{{\sum\:}_{j=1}^{n}v\left({d}_{j}\right)}$$

$$\:v\left({d}_{j}\right)=0.5exp\left(\frac{{d}_{j}^{2}}{{p}_{k}{D}^{2}}\right)$$.

where.

- $\:A\left({s}_{j}\right)$ is the daily rainfall anomaly observed from a reference station located at $\:{s}_{j}$,
- $\:{d}_{j}$ is the distance between the target location $\:s$ and the reference station $\:{s}_{j}$,
- $\:{g}_{k-1}\left(s\right)$ and $\:{g}_{k-1}\left({s}_{j}\right)$ are the ($\:k-1)$-th pass Barnes spatial interpolation at the target location $\:s$ and the reference station $\:{s}_{j}$, respectively,
- $\:D=200km$ is a width parameter,
- $\:{p}_{0}=1$ and $\:{p}_{1}=0.4$ are two smoothing parameters.

**Fig. 9:**
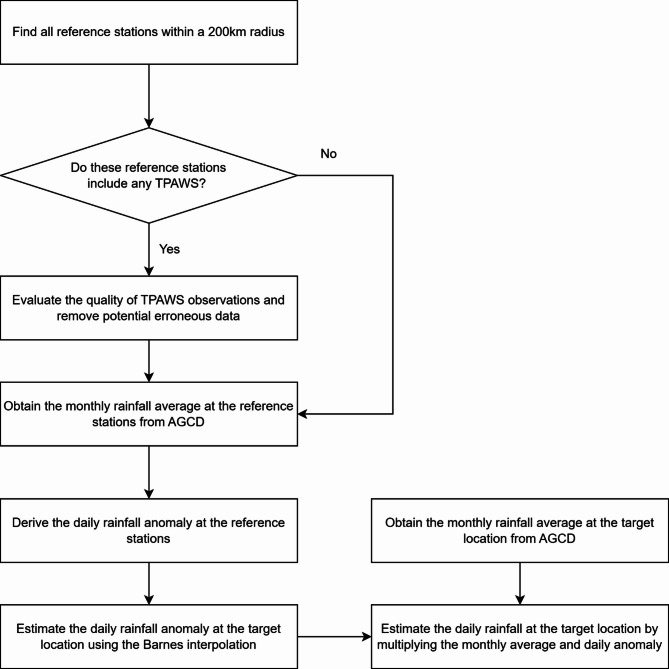
Flowchart of the rainfall estimation process incorporating TPAWS observations.

The Barnes interpolation method offers several advantages, including computational efficiency with Fast Barnes Interpolation algorithm developed by Zürcher^31^, robustness in handling strong gradients and areas with missing data, flexibility in allowing parameters to be adjusted. Previous research^32,33^ has demonstrated its comparable accuracy to more complex techniques while avoiding some disadvantages such as unrealistic extrapolated values and reduced variability. Figure 9 illustrates the proposed daily rainfall estimation through a flow chart.

### Network density calculation

To assess DPIRD station influence on the accuracy of rainfall estimates, network density for a specific validation station located at $\:s$ is calculated as:

$$\:\rho\:\left(s\right)={\sum\:}_{i=1}^{n}\text{exp}\left\{-\frac{1}{2}{\left[\frac{d\left(s,{s}_{i}\right)}{10}\right]}^{2}\right\}$$

where $\:d\left(s,{s}_{i}\right)$ is the distance to a DPIRD station located at $\:{s}_{i}$ in kilometres. Nipen, et al.^34^ also interpreted this measure as the weighted number of stations within 10 km.

## Electronic supplementary material

Below is the link to the electronic supplementary material.

Supplementary Material 1

## Data Availability

BoM station data were obtained from the BoM Climate Data Online (Bureau of Meteorology, 2022). DPRID station data were accessed via an Application Programming Interface (API) (DPIRD, 2022). AGCD data are available from the BoM Climate Data Online (Bureau of Meteorology, 2022) or through the National Computational Infrastructure (NCI) data collections (NCI, 2022). The datasets used and/or analysed in this study are available from the corresponding author on reasonable request.
