# Supplementary material for: Enhancing gridded climate products with third party weather data in a rainfall study from Western Australia
Source: Sci Rep. 2025 Oct 21;15:36522. doi: 10.1038/s41598-025-11145-0 (PMC12540729; doi:10.1038/s41598-025-11145-0)
Supplement: Supplementary file 1 — Supplementary Material 1 [file 41598_2025_11145_MOESM1_ESM.pdf]

Supplementary material for “Enhancing Gridded Climate Products with Third Party Weather Data in a Rainfall Study from Western Australia”

Ming Li[1*] and Quanxi Shao[1]

[1]CSIRO Data61, PO Box 1130, Bentley, WA 6102, Australia

*Corresponding author:

Ming Li

Tel: +61-8-9333 6417

Fax: +61-8-9333 6121

Email: Ming.Li@data61.csiro.au

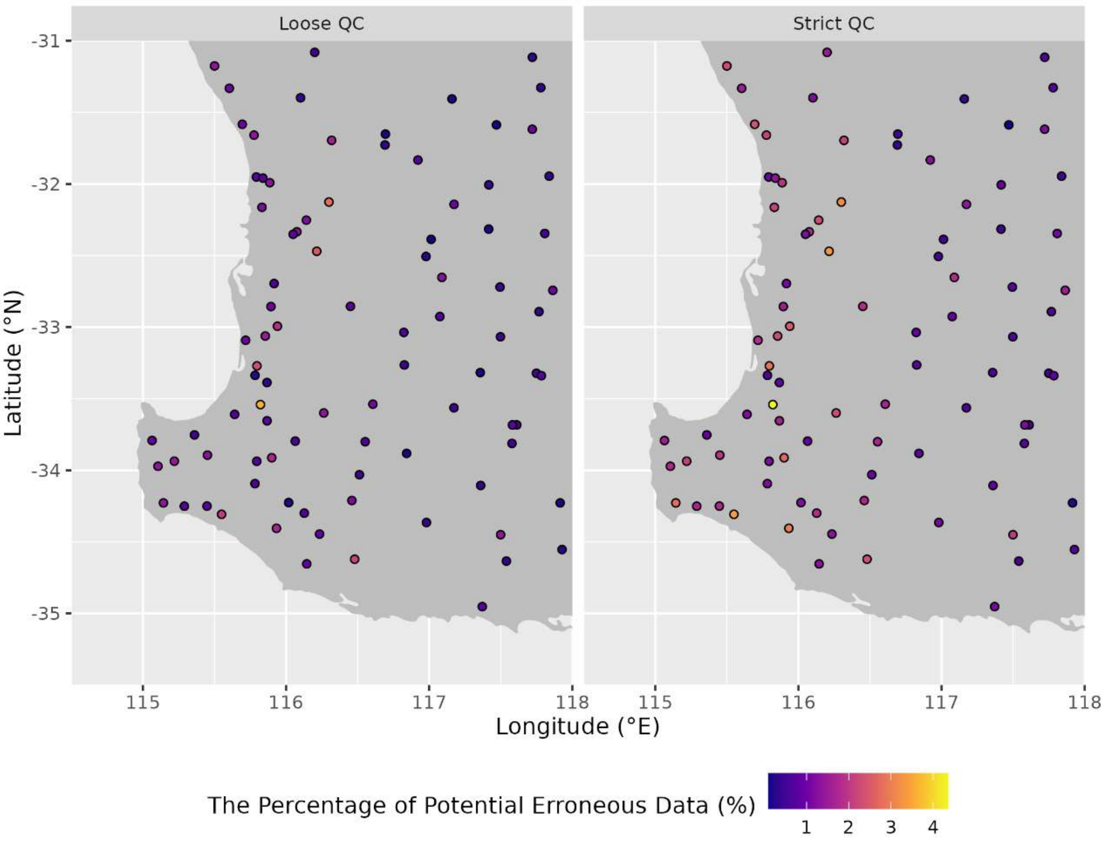

Figure S1: Spatial distribution of the station-wise potential erroneous data rate for DPIRD stations under loose and strict QC conditions.

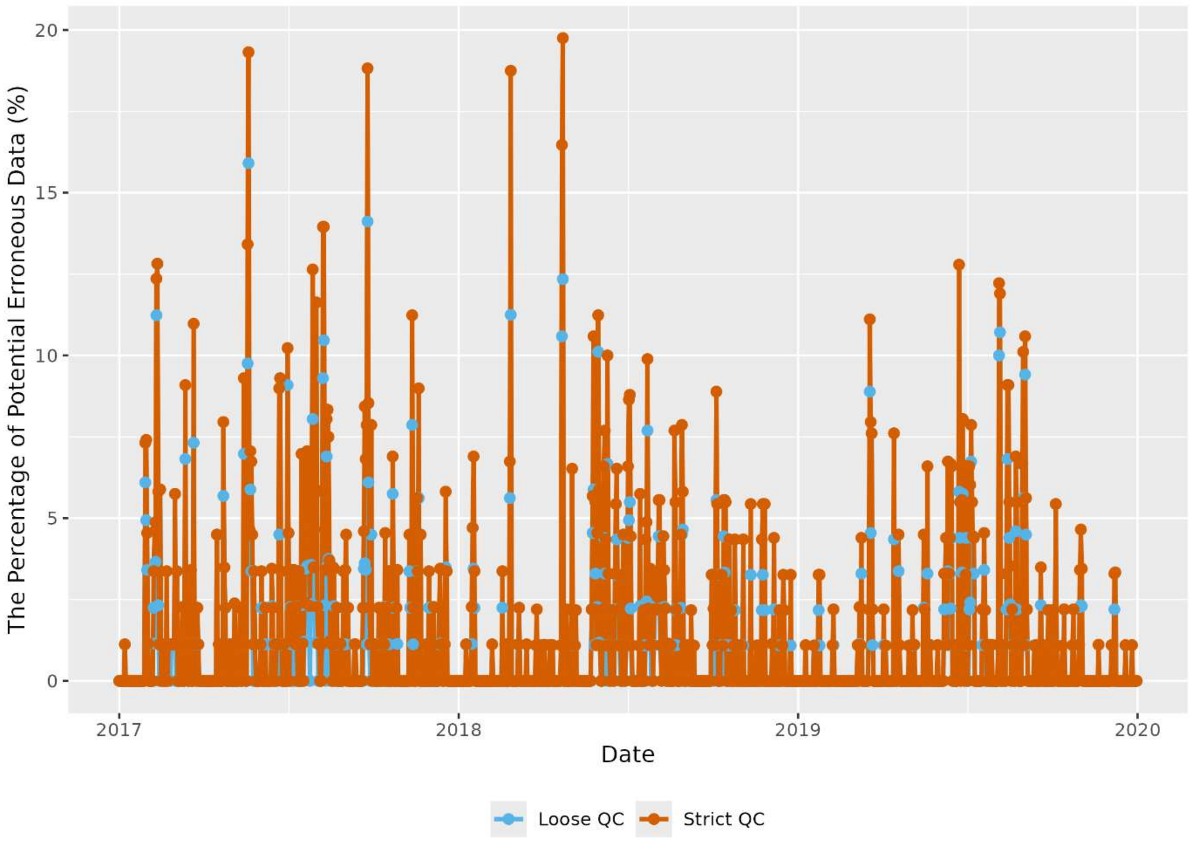

Figure S2: Time series of the percentage of potential erroneous data across the selected 95 DPIRD stations under loose and strict QC conditions.

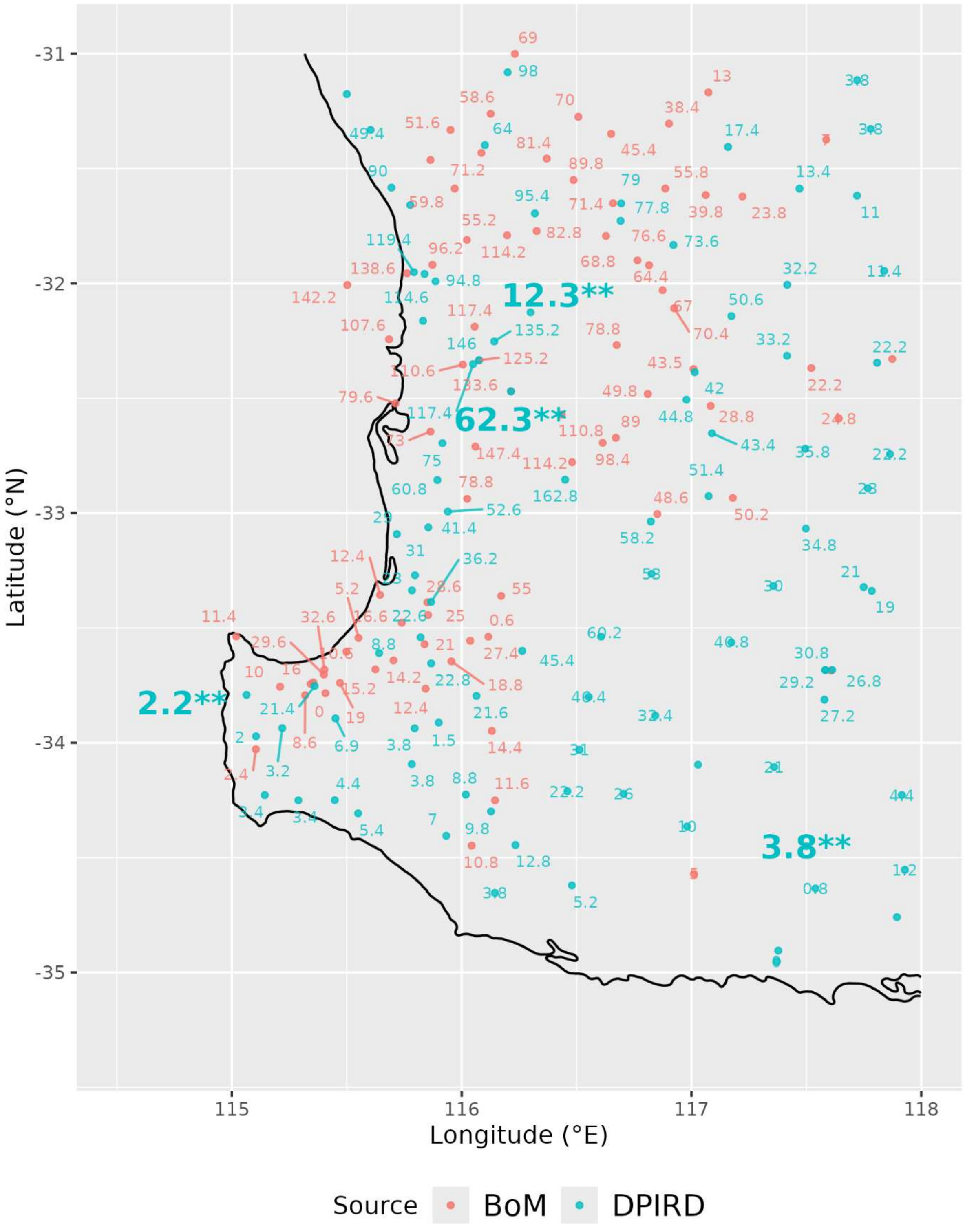

Figure S3: Map of rainfall observations from BoM and DPIRD stations for 16/01/2018. Possible erroneous observations from DPIRD stations, identified by loose quality control (double asterisks) and strict quality control (single asterisk), are highlighted in large font.

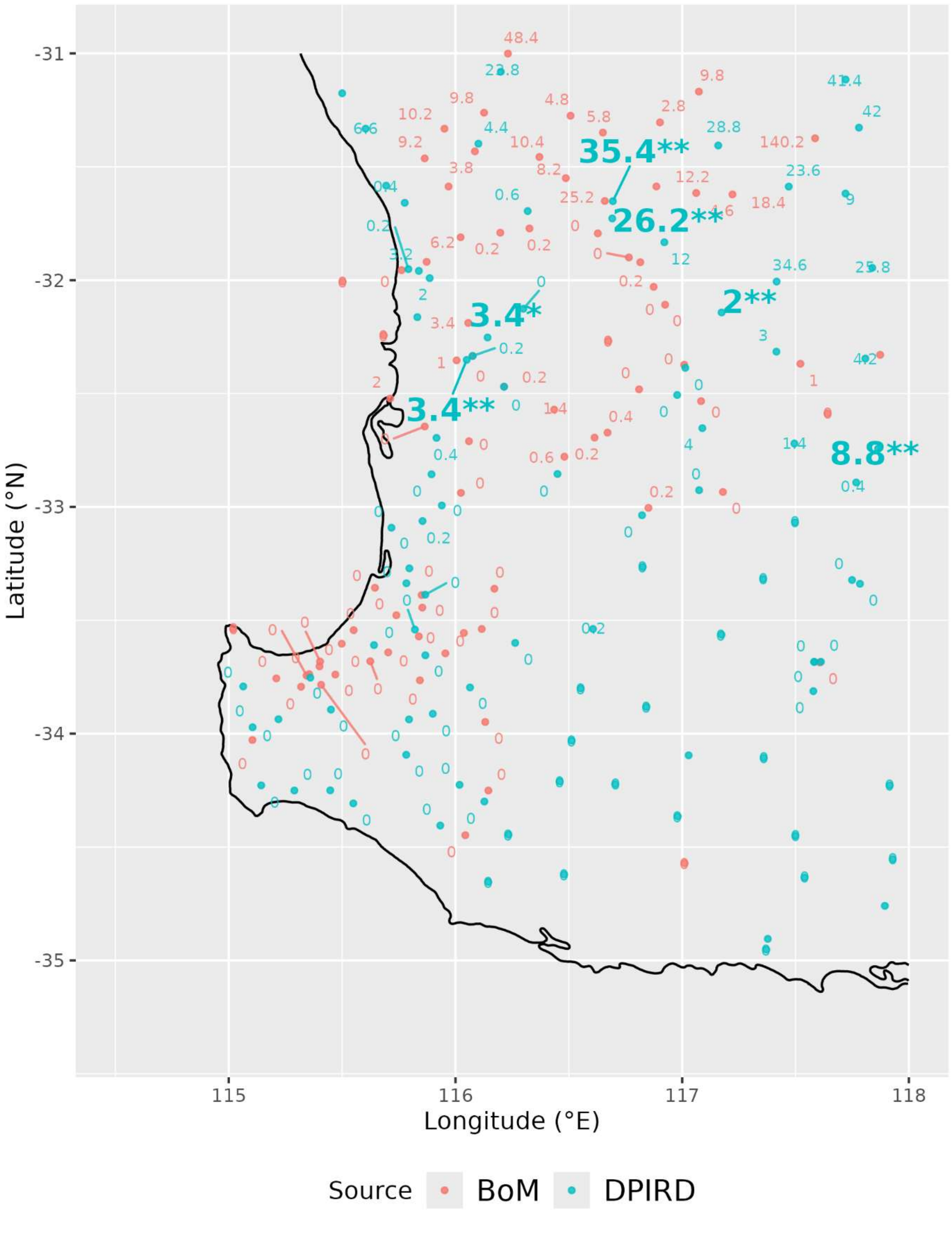

Figure S4: Map of rainfall observations from BoM and DPIRD stations for 29/01/2017. Possible erroneous observations from DPIRD stations, identified by loose quality control (double asterisks) and strict quality control (single asterisk), are highlighted in large font.

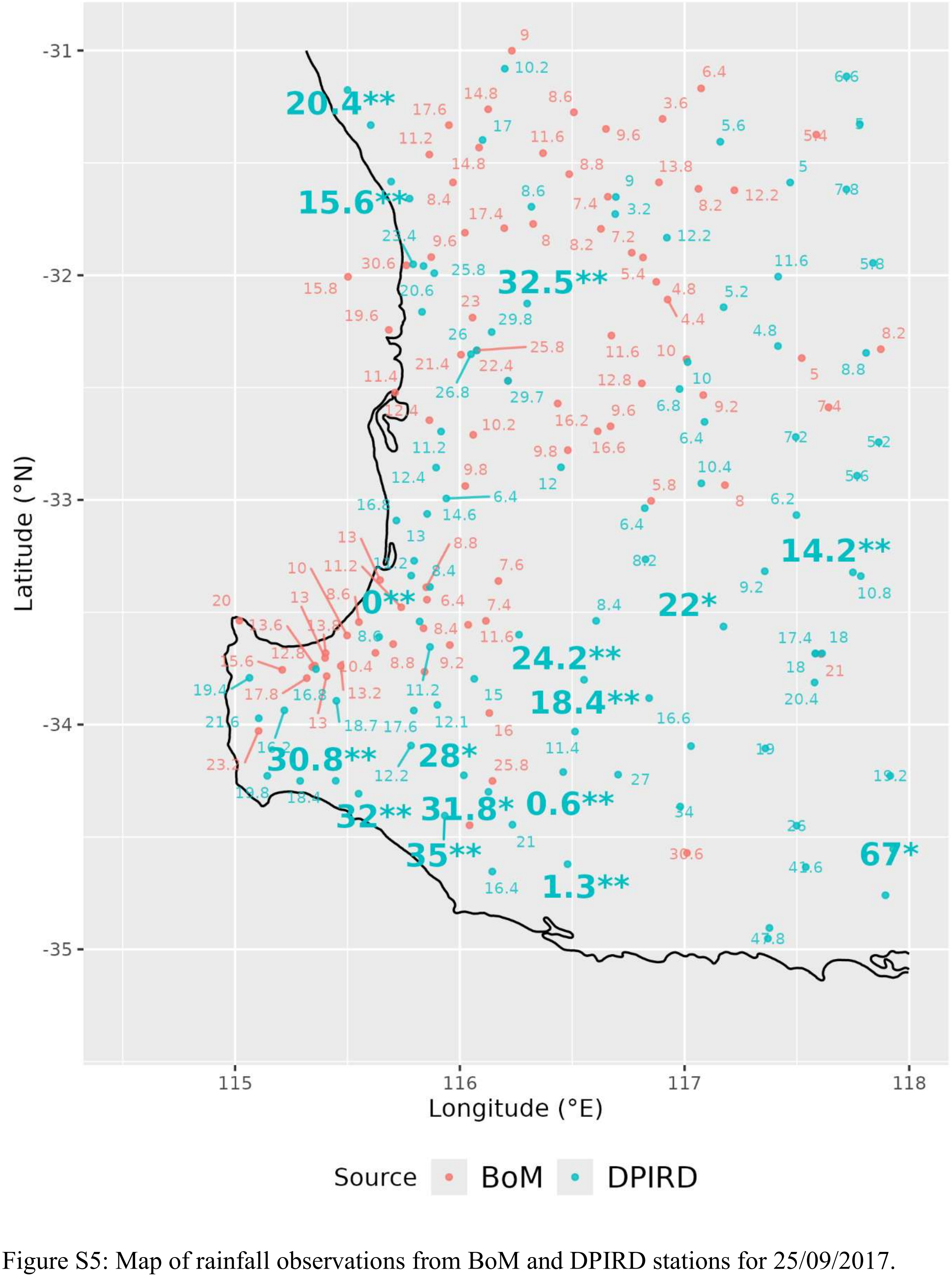

Figure S5: Map of rainfall observations from BoM and DPIRD stations for 25/09/2017. Possible erroneous observations from DPIRD stations, identified by loose quality control (double asterisks) and strict quality control (single asterisk), are highlighted in large font.
